# Comparable gut mucosal breakdown and microbial translocation in severe COVID-19 with and without HIV infection

**DOI:** 10.1097/QAD.0000000000004166

**Published:** 2025-05-08

**Authors:** Sabrina Marozin, Valeria Bono, Martina Mazzoccoli, Matteo Augello, Roberta Rovito, Lucia Taramasso, Antonio Di Biagio, Annapaola Callegaro, Franco Maggiolo, Elisa Borghi, Camilla Tincati, Giulia Marchetti

**Affiliations:** a Clinic of Infectious Diseases and Tropical Medicine, San Paolo Hospital, ASST Santi Paolo e Carlo, Department of Health Sciences, University of Milan; b Laboratory of Microbiology and Clinical Microbiology, Department of Health Sciences, University of Milan, Milan; c Infectious Diseases Unit, San Martino Policlinico Hospital; d Department of Health Sciences, University of Genoa, Genoa; e Biobank Unit and Microbiology and Virology Laboratory; f Division of Infectious Diseases, ASST Papa Giovanni XXIII, Bergamo, Italy.

## Abstract

In this study, we explored the link between gut impairment and severe COVID-19 in people with HIV (PWH). We compared PWH with COVID-19 to HIV-uninfected individuals hospitalized for the same condition. Despite more severe clinical symptoms in PWH, we did not observe significant differences in gut barrier dysfunction markers between the two groups. Our findings suggest a link between SARS-CoV-2 pneumonia and gut damage, independent of HIV status, warranting further investigation into its role in COVID-19 severity.

The impact of HIV as a risk factor in the morbidity of COVID-19, especially early in the pandemic, has been long debated. Several data indicate that the severity of the clinical outcome of SARS-CoV-2 infection in people with HIV (PWH) under combination antiretroviral treatment (cART) is equivalent to people without HIV [1,2]. Yet, progression to more severe COVID-19 and its related complications have been observed in PWH, in association with comorbidities, low CD4^+^ cell count and detectable viral load [3,4]. Impaired intestinal barrier function as well as subsequent microbial translocation, are considered pathogenic hallmarks of HIV infection and disease progression [5]. Of note, the same features have been linked to the development of severe SARS-CoV-2 infection also in PWH [6].

We, therefore, hypothesized that gut damage/microbial translocation represent an underlying cause of severe COVID-19 in PWH.

To this end, we enrolled 16 PWH and 18 sex-matched and age-matched HIV-uninfected individuals, for whom frozen plasma samples were available, who sought medical care for acute COVID-19 during the initial SARS-CoV-2 pandemic in 2020–2021.

Individuals with COVID-19 were recruited during the first wave of the pandemic (March–May 2020), whereas PWH-COVID-19 participants were enrolled during the second wave (October 2020–April 2021). Neither study group had received the SARS-CoV-2 vaccine at the time of study. All individuals were hospitalized for COVID-19-related pneumonia. SARS-CoV-2 was confirmed by positive nasopharyngeal swab (RT-PCR) and radiological imaging of pneumonia. Plasma was used to determine levels of microbial translocation [lipopolysaccharide (LPS)-binding protein (LBP), Hycult Biotech kit], microbial-driven monocyte activation [soluble cluster of differentiation 14 (CD14), Hycult Biotech kit], gut damage [intestinal fatty-acid binding protein (I-FABP), Hycult Biotech kit] and structure [Epithelial cadherin (E-Cadherin); tight junction protein Zonulin, Elabscience kit] by ELISA, following the manufacturer's instructions. Descriptive and statistical analyses were performed with the use of GraphPad Prism 9.0 (GraphPad Inc., La Jolla, California, USA). Categorical variables were reported as percentages (Fisher's exact test) and continuous variables were reported as median (interquartile range, IQR). Comparisons among groups were performed by Mann–Whitney *U* test.

The two groups were comparable for age, sex, ethnicity and major comorbidities. Regarding COVID-19 symptomatology, PWH more frequently reported cough *(P* < 0.0001), presented a later admission to the hospital from the onset of the symptoms (9.5 versus 5.5 days, *P* = 0.023), had a lower PaO_2_/FiO_2_ nadir and were more frequently treated with mechanical ventilation (31.2 versus 16.7%, *P* *=* 0.02) as well as medical therapy during hospitalization (*P* < 0.0001) compared to non-PWH (*P* = 0.0001). The median duration of symptoms before biological sample collection was 5.5 days in individuals with COVID-19 and 9.5 days in PWH-COVID-19. The clinical outcome was comparable between groups (Table S1).

The majority of PWH (87.5%) were on cART and 81.2% had undetectable HIV RNA (<50 copies/ml). CD4^+^ T-cell nadir was 59 (IQR 12.5– 331.3 cells/μl), current CD4^+^ T-cell count and CD4^+^/CD8^+^ ratio were, respectively, 395 cells/μl (IQR 171–776 cells/μl) and 0.4 (IQR 0.17–1.05).

We observed a trend to higher levels of LBP in PWH-COVID-19 than individuals with COVID-19 alone [21677 ng/ml (IQR: 8447–28552) versus 16408 ng/ml (IQR: 7654–23 415); *P* = 0.09; Fig. 1a]. In contrast, sCD14 [2563 ng/ml (IQR: 1728–3587) versus 1807 ng/ml (IQR: 1757–2398); *P* = 0.4; Fig. 1b), I-FABP [128.8 pg/ml (49–502) versus 183.3 pg/ml (114–360.5); *P* = 0.4; Fig. 1c], E-Cadherin [167 ng/ml (128–196) versus 149.7 ng/ml (121–173); *P* = 0.3; Fig. 1d] and Zonulin [119 ng/ml (40–175) versus 146.6 ng/ml (52–147); *P* = 0.8; Fig. 1e] were comparable in the two groups.

**Fig. 1:**
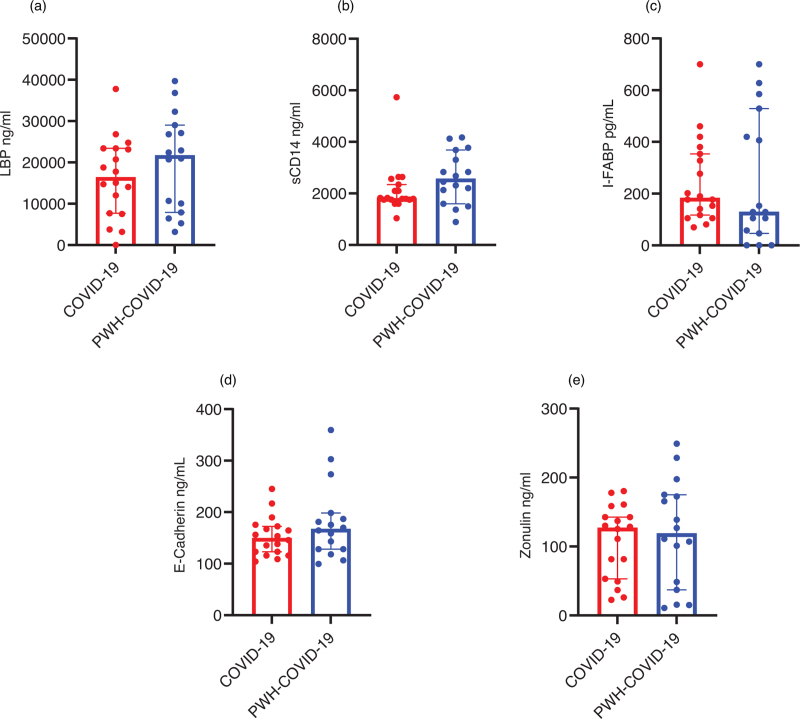
(a–e) Levels of intestinal barrier permeability and microbial translocation during severe COVID-19 in non-PWH (COVID-19) and PWH (PWH-COVID-19).

Serum concentrations of lipopolysaccharide (LPS)-binding protein (LBP); soluble cluster of differentiation 14 (CD14); intestinal fatty-acid binding protein (I-FABP); epithelial cadherin (E-Cadherin); tight junction protein (Zonulin). Statistical analysis: Mann–Whitney *U* test. PWH, people with HIV.

The present research was conducted to assess whether gut barrier breakdown, a known feature of both HIV and SARS-CoV-2 infection, might be a factor contributing to severe COVID-19 in PWH. We hypothesized higher levels of microbial translocation/mucosal barrier dysfunction markers in PWH hospitalized for COVID-19 compared to those without HIV. Indeed, previous studies report that LBP, sCD14, I-FABP, E-cadherin, and Zonulin levels are generally lower in healthy controls compared to those seen in pathological conditions involving systemic inflammation or intestinal barrier dysfunction [7–9]. Surprisingly, despite a tendency to a more severe clinical presentation of COVID-19 in PWH, we did not detect significant differences in LBP, sCD14, I-FABP, E-cadherin, and Zonulin levels between the two groups. A possible explanation for our findings is that PWH with COVID-19 pneumonia were more frequently treated with antiviral and steroid therapy, which may have contained, at least in part, SARS-CoV-2-mediated damage in the gut.

Our study has several drawbacks, including the lack of non-COVID-19 controls, its cross-sectional design and small sample size; indeed, the present work was conducted as a pilot study to investigate the trends in markers of gut function/damage and generate hypotheses for future research. Furthermore, Zonulin measurement by ELISA might have been affected by interference from albumin and complement components, as reported by recent research [10]. Despite these limitations, our research highlights that gastrointestinal impairment is similar between the two study groups, regardless of HIV status, and may therefore be linked to critical COVID-19 condition. Future studies should focus on assessing the long-term kinetics of gut injury and changes in microbiota composition in individuals with severe COVID-19 and better understand its possible role in the development of postacute sequelae of SARS-CoV-2 infection (PASC) [11,12].

## Acknowledgements

This study was supported by funding from the European Union‘s Horizon 2020 Research and Innovation Program under grant agreement no. 101016167 within the ORCHESTRA project (Connecting European Cohorts to Increase Common and Effective Response to SARS-CoV-2 Pandemic), and grant agreement no. 101046016 within the EuCARE project (European Cohorts of Patients and Schools to Advance Response to Epidemics).

We thank Miss Maria Sgarlata and Dr Silvia Ancona for technical support.

We are grateful to all the patients and the contributors, whose effort made this small study possible.

### Conflicts of interest

There are no conflicts of interest.

## Supplementary Material

Supplemental Digital Content
