# Supplementary material for: Comparable gut mucosal breakdown and microbial translocation in severe COVID-19 with and without HIV infection
Source: AIDS. 2025 May 8;39(7):921–3. doi: 10.1097/QAD.0000000000004166 (PMC12077341; doi:10.1097/QAD.0000000000004166)
Supplement: Supplemental Digital Content [file aids-39-921-s001.docx]

**Supplementary material**

**Table S1.** Cohort demographic and clinical information of PWH (People With HIV) hospitalized for acute SARS-CoV-2 infection (PWH-COVID-19) and non-PWH with SARS-CoV-2 (COVID-19).

|  | Study population  (n=34) | COVID-19  (n=18) | PWH-COVID-19  (n=16) | *P* value |
| --- | --- | --- | --- | --- |
|  |  |  |  |  |
| Age, median yr (IQR) | 57 (46-65) | 57 (45-64) | 57 (46-65) | 0.8 |
| Male sex, no. (%) | 28 (82.3) | 15(83.3) | 13 (81.2) | >0.1 |
| Ethnicity, no (%) |  |  |  | 0.35 |
| Caucasian | 28 (82.3) | 15 (83.3) | 13 (81.25) |  |
| Latin-American | 4 (11.8) | 3 (16.7) | 1 (6.25) |  |
| African | 2 (5.9) | 0 | 2 (12.5) |  |
| Comorbidities, no (%) |  |  |  |  |
| Hypertension | 14 (41.2) | 7 (38.9) | 7 (43.8) | >0.1 |
| Chronic heart disease | 2 (5.9) | 0 (0) | 2 (12.5) | 0.21 |
| Chronic pulmonary disease | 3 (8.8) | 2 (11.1) | 1 (6.25) | >0.1 |
| Chronic kidney disease | 2 (5.9) | 0 (0.0) | 2 (12.5) | 0.21 |
| Diabetes | 2 (5.9) | 1 (5.6) | 1 (6.25) | >0.1 |
| Symptoms at hospital admission, no % |  |  |  |  |
| Fever | 31 (91.2) | 18 (100) | 13 (81.2) | 0.045 |
| Fatigue | 19 (55.9) | 13 (72.2) | 6 (37.5) | 0.082 |
| Cough | 19 (55.9) | 16 (88.9) | 3 (18.7) | <0.0001 |
| Dyspnea | 19 (55.9) | 9 (50) | 10 (62.5) | >0.1 |
| Anosmia/dysgeusia | 16 (47.1) | 11 (61.1) | 5 (31.2) | 0.10 |
| PaO2/FiO2 nadir, median (IQR) | 176 (140-347) | 342 (211-377) | 140 (122-151) | 0.0001 |
| Duration of symptoms before biological sample collection, days (median, IQR) | 6.5 (4-10) | 5.5 (3-8.5) | 9.5 (6.5-12.50) | 0.023 |
| Reported GI symptoms, no. (%) | 11 (32.3) | 7 (38.9) | 4 (25) | 0.42 |
| Maximum oxygen therapy |  |  |  | 0.02 |
| Non-mechanical ventilation | 21 (61.8) | 11 (61.1) | 10 (62.5) |  |
| CPAP/NIV/OTI | 8 (23.5) | 3 (16.7) | 5 (31.25) |  |
| Ambient air | 5 (14.7) | 4 (22.2) | 1 (6.25) |  |
| Treatment, no (%) |  |  |  | <0.0001 |
| Steroids | 9 (26.5) | 0 (0.0) | 9 (56.2) |  |
| Remdesivir | 6 (17.6) | 0 (0.0) | 6 (37.5) |  |
| Outcome, no (%) |  |  |  | 0.4706 |
| Death | 1 (2.9) | 0 (0.0) | 1 (6.3) |  |
| Dismissal | 34 (97.1) | 18 (100) | 15 (93.7) |  |
